# Complete Response of High Microsatellite Instability Gastric Cancer and Synchronous Microsatellite Stability Rectal Cancer

**DOI:** 10.7759/cureus.25820

**Published:** 2022-06-10

**Authors:** Zachary E Hunzeker, Pooja Bhakta, Sindusha R Gudipally, Sri Bharathi Kavuri, Rohit Venkatesan, Chukwuyejulumafor Nwanze

**Affiliations:** 1 Medicine, University of Texas Medical Branch, Galveston, USA; 2 Internal Medicine, University of Texas Medical Branch, Galveston, USA; 3 Oncology, University of Texas Medical Branch, Galveston, USA; 4 Pathology, University of Texas Medical Branch, Galveston, USA; 5 Oncology, University of Texas MD Anderson Cancer Center, Houston, USA

**Keywords:** microsatellite instability, colorectal cancer, immunotherapy, gastric cancer, rectal cancer

## Abstract

Gastric cancer, a leading cause of cancer-related death in the world, may occur with an additional synchronous malignancy in rare cases. Of these rare cases many are colorectal cancer. Microsatellite instability is a phenomenon that may contribute to the pathogenesis of both cancers, as are field cancerization and genetic susceptibility, although none of these explain many concurrent cases. In this case, we described a patient with locally advanced microsatellite instability-high gastric cancer and synchronous microsatellite stable rectal cancer, who received a combination chemo-immunotherapy regimen and achieved complete response. This report reflects on current knowledge surrounding synchronous primary malignancies and achieving complete response.

## Introduction

Additional primary malignancies (APMs) have been reported in up to 10.9% of gastric cancer cases and 10.2% of colorectal cancer cases [1,2]. Multiple primary malignancies (MPMs) are associated with poor overall survival compared to metachronous malignancies. Additional primary malignancies (APMs) in gastric cancer patients have been shown to lead to over 30% lower 10-year overall survival rates relative to solitary gastric cancer, although this study did not stratify for stage [1]. Regardless of the mechanism contributing to higher rates of multiple malignancies, this high incidence warrants physicians to consider the possibility of an APM in all cancer patients, and consistent long-term follow-up is essential.

Gastric cancer is the third most common cause of cancer-related deaths worldwide on its own [3]. When caught early, gastric cancer can be treated with endoscopic dissection, shown to lead to three- and five-year overall disease-free survival rates of over 95% [3]. In addition to radical surgical resection, available treatment options for advanced gastric cancer include neoadjuvant chemotherapy, radiation, various molecular-targeted therapies, and most recently, immunotherapy.

The role of immunotherapy in gastric cancer is an active area of interest, but progress has been made in identifying predictive markers for immunotherapy response. Microsatellite instability-high (MSI-H) status, Epstein-Barr virus-positive status, and high tumor mutational burden have emerged as molecular markers associated with high tumor microenvironment programmed death-ligand 1 (PD-L1) expression and/or leukocyte infiltration, enabling immunotherapy success, particularly at the site of PD-1/PD-L1 interaction [4]. Microsatellite instability refers to the extent of variation in hypermutable repeating DNA motifs scattered throughout the tumor genome, often owing to a defect in the mismatch repair (MMR) pathway [4]. MSI-H status is estimated to be prevalent in 19% of gastric cancers [5]. Nivolumab, a monoclonal antibody to PD-1, recently received FDA approval for the treatment of advanced gastric cancer when used alongside folinic acid, fluorouracil, and oxaliplatin (FOLFOX) chemotherapy [6,7]. In this case report, we reported a patient with a primary microsatellite instable (MSI-H) gastric cancer with co-existing primary microsatellite stable (MSS) rectal cancer, requiring multimodal treatment involving immunotherapy, chemotherapy, and surgery. Nivolumab with FOLFOX was decided upon, which in addition to surgical resection achieved near complete response for both primary malignancies [7].

## Case presentation

**Figure 1:**
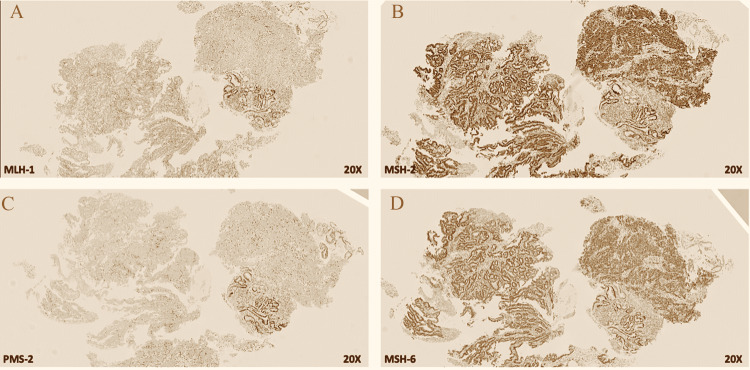
Gastric adenocarcinoma immunohistochemistry shows high microsatellite instability with loss of both MLH-1 and PMS-2 (MSI-High) (A and C) and increased enhancement in MLH-2 and MSH-6 (B and D).

**Figure 2:**
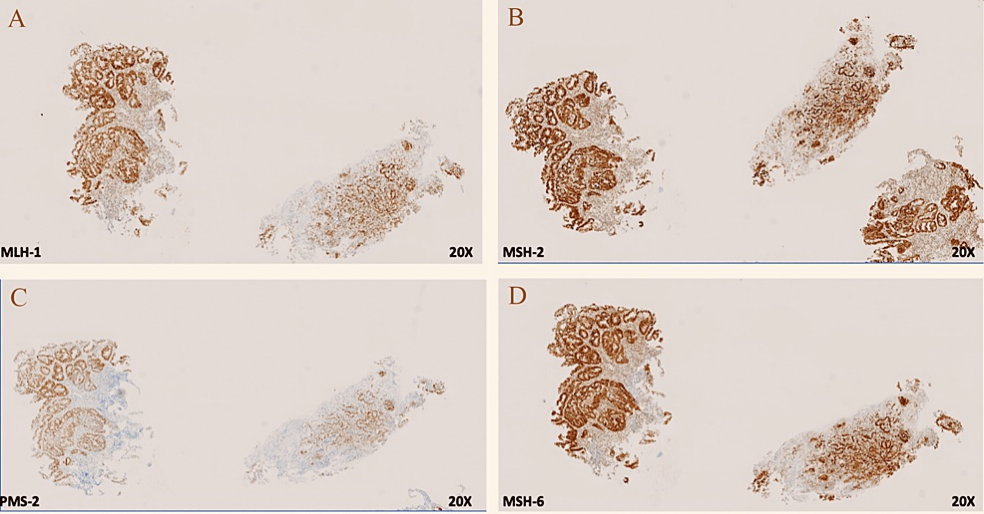
Rectal adenocarcinoma immunohistochemistry shows microsatellite stability with intact staining of MLH-1, PMS-2, MSH-2, and MSH-6. There is positive enhancement in staining in MLH-1, PMS-2, MSH-2, and MSH-6 (A-D).

**Figure 3:**
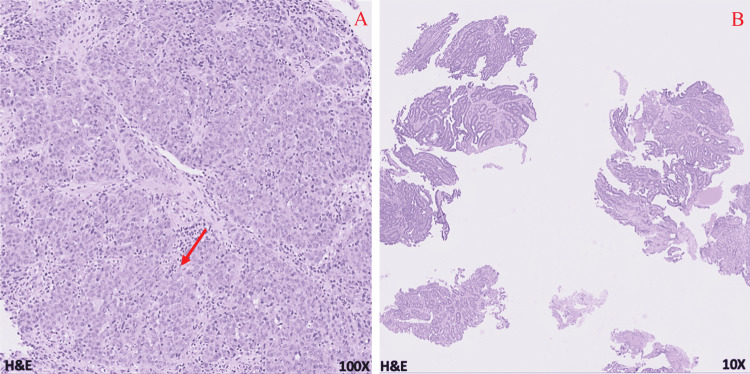
Gastric adenocarcinoma microscopic sections show areas of moderately differentiated gastric adenocarcinoma (arrow) (A) with adjacent benign gastric epithelium (B).

**Figure 4:**
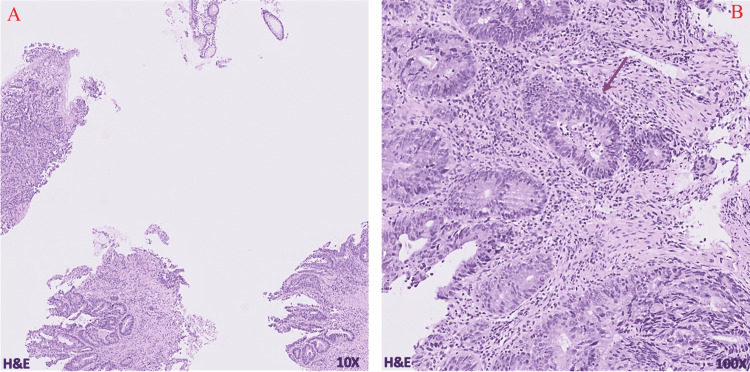
Rectal adenocarcinoma microscopic sections show areas of moderately differentiated rectal adenocarcinoma (A) with foci of comedonecrosis (arrow) (B).

A 69-year-old male with a 37.5-pack-year smoking history presented initially to urgent care with a three-month history of constant epigastric and abdominal pain as well as an 18-pound weight loss. Further workup by gastroenterology with endoscopy revealed a large, fungating, circumferential mass with bleeding in the gastric antrum/pylorus. Concurrent colonoscopy also found a 30 mm polypoid semi-sessile lesion in the proximal rectum among other smaller polyps ranging from 4 to 20 mm. Immunohistochemical staining (IHC) of the biopsies showed patchy positive cytokeratin 7 (CK7), mostly negative CK20, diffusely positive CDX2 signal in the gastric tumor; and negative CK7, patchy positive CK20, diffusely positive CDX2 signal in the rectal tumor (Figures 1A-1D, 2A-2D). This result was consistent with separate primary gastric adenocarcinoma and primary colorectal adenocarcinoma (Figures 3A, 3B, 4A, 4B). Microsatellite instability (MSI) testing showed MLH1(-), PMS2(-), MSH2(+), MSH6(+) expression pattern in the gastric tumor consistent with microsatellite instability-high (MSI-H) status, versus MLH1(+), PMS2(+), MSH2(+), MSH6(+) expression in the rectal tumor consistent with microsatellite stable (MSS) status. The rectal sample was negative for NRAS, BRAF, and KRAS mutations.

After discussion at the multidisciplinary tumor board, the patient proceeded to receive neoadjuvant FOLFOX with nivolumab as per the CheckMate 649 trial [7]. The rationale behind selecting this regimen was that the MSI high gastric adenocarcinoma may respond well to immunotherapy while the microsatellite stable rectal adenocarcinoma may respond to FOLFOX. Patient tolerated four cycles well with significant improvement in clinical symptoms including better ability to tolerate oral intake leading to weight gain. Follow-up CT thorax and abdomen/pelvis was performed after four cycles, three months after initial imaging, showing no measurable gastric tumor and no evidence of the rectal tumor. MR pelvis was also performed at this time which further found no perceivable rectal mass and no perirectal or pelvic lymphadenopathy. Repeat esophagogastroduodenoscopy (EGD) and colonoscopy by GI found no evidence of residual carcinoma and subsequent biopsy was also negative for malignancy.

He subsequently underwent a partial gastrectomy with D2 lymph node dissection and gastrojejunostomy and small bowel resection. Pathology revealed R0 resection with all resected lymph nodes negative for malignancy and small T2 residual cancer making the final pathologic stage ypT2ypN0. The patient went on to receive four additional cycles of FOLFOX and nivolumab in the post-operative setting and remains in radiographic and endoscopic remission at the time of this writing.

## Discussion

Synchronous tumors are primary malignancies that present within six months of another tumor. Gastric cancer has been found to have associated primary tumors in 2.4-10.9% of cases, with one study finding that 0.9% of all gastric cancers coexist with a synchronous primary non-gastric malignancy [1,8]. This presents a challenge for treatment, of which there is no standard in this specific area. Surgery and adjuvant therapy may be the most common to perform [9]. While prognosis is poorer, it may be largely dependent on the stage of the involved cancers [10-12].

The mechanism of multiple synchronous primary malignancy development is still unclear, but genetic susceptibility, field cancerization, and microsatellite instability are possible factors at play. Genetic susceptibility should be considered particularly in younger patients with MPMs and a family history of cancer, and field cancerization is especially more likely when MPMs are in proximity and of the same tissue type (i.e., colon and rectal cancer). A five-marker mononucleotide and dinucleotide repeat polymerase chain reaction (PCR) test has been developed with established guidelines for diagnosing MSI, which should be performed in any patient with MPMs, particularly of colon and/or endometrial origins [13]. Prior studies of microsatellite instability-associated APM development have shown varying degrees of MSI status across the multiple primary malignancies. Universal MSI-H status of gastrointestinal cancers seems to be more prevalent in synchronous tumors of the same organ (i.e., multiple primary gastric tumors) while heterogeneous MSI status appears more common in tumors of different organs [14,15]. This is in line with our presented patient. Nonetheless, having any MSI-H tumors requires a unique approach to treatment.

While rectal cancer has been shown to respond well to chemotherapy with oxaliplatin-based regimens, MSI-H gastric cancer is generally chemo-resistant [16-18]. However, it has been shown to be more vulnerable to immunotherapy treatment, with PD-1 blockade [4]. The CheckMate 649 trial has shown that nivolumab with chemotherapy improves overall survival in gastric cancer compared to chemotherapy alone [7]. Given the presumed benefit of PD-1 blockade for MSI-H gastric cancer and obvious necessity for chemotherapy in our patient with coexisting rectal cancer, we treated with a regimen of FOLFOX and nivolumab.

While the most common APM with primary gastric cancer is colorectal cancer, synchronous gastric and rectal adenocarcinomas have still rarely been reported, and MSI-H status involvement is even less so [1,19,20]. Herein, we presented a patient with MSI-H gastric adenocarcinoma and synchronous MSS rectal adenocarcinoma, who achieved a complete response in rectal adenocarcinoma as well as R0 resection and very good response in gastric cancer after partial gastrectomy and eight total cycles of chemoimmunotherapy combination regimen. This outcome reinforces the need for microsatellite testing in gastric cancers, as well as the current treatment regimens for multiple primary malignancies and MSI-H gastric cancer. The model approach of neoadjuvant or adjuvant treatment with surgical resection for multiple primary malignancies was applied to this patient with success. The concomitant use of nivolumab with FOLFOX resulted in significant response in ability to achieve R0 resection in this patient’s gastric cancer. While the extent to which nivolumab and FOLFOX individually affected this patient’s gastric tumor is unknown, it remains a possibility that nivolumab-augmented chemotherapy regimens may be of more benefit in chemo-resistant MSI-H gastric cancer in the pre-operative setting compared to immunotherapy alone. However, further studies on this topic are likely necessary.

## Conclusions

We would like to highlight the value of microsatellite testing in gastric and rectal tumors and the efficacy of Nivolumab in the setting of MSI-H disease. MPMs are unexpected for physicians to encounter but may still be manageable in the setting of rapidly progressing cancer molecular characterization and treatment. This case report adds to the current knowledge about treating multiple, heterogeneous microsatellite expression cancers, as well as reinforces recent and ongoing studies into PD-1 blockade for MSI-H gastric cancer.

## References

[1] Ikeguchi M, Ohfuji S, Oka A, Tsujitani S, Maeda M, Kaibara N (1995). Synchronous and metachronous primary malignancies in organs other than the stomach in patients with early gastric cancer. Hepatogastroenterology.

[2] Jia H, Li Q, Yuan J, Sun X, Wu Z (2020). Second primary malignancies in patients with colorectal cancer: a population-based analysis. Oncologist.

[3] Okada K, Fujisaki J, Yoshida T (2012). Long-term outcomes of endoscopic submucosal dissection for undifferentiated-type early gastric cancer. Endoscopy.

[4] Kwak Y, Seo AN, Lee HE, Lee HS (2020). Tumor immune response and immunotherapy in gastric cancer. J Pathol Transl Med.

[5] Hause RJ, Pritchard CC, Shendure J, Salipante SJ (2016). Classification and characterization of microsatellite instability across 18 cancer types. Nat Med.

[6] (2022). Targeted therapy directed by genetic testing in treating patients with advanced refractory solid tumors, lymphomas, or multiple myeloma (The MATCH Screening Trial). https://clinicaltrials.gov/ct2/show/NCT02465060.

[7] Janjigian YY, Shitara K, Moehler M (2021). First-line nivolumab plus chemotherapy versus chemotherapy alone for advanced gastric, gastro-oesophageal junction, and oesophageal adenocarcinoma (CheckMate 649): a randomised, open-label, phase 3 trial. Lancet.

[8] Dinis-Ribeiro M, Lomba-Viana H, Silva R, Moreira-Dias L, Lomba-Viana R (2002). Associated primary tumors in patients with gastric cancer. J Clin Gastroenterol.

[9] Irimie A, Achimas-Cadariu P, Burz C, Puscas E (2010). Multiple primary malignancies--epidemiological analysis at a single tertiary institution. J Gastrointestin Liver Dis.

[10] Sert F, Caner A, Haydaroglu A (2020). Trends in the incidence and overall survival of multiple primary cancers in Turkey. J BUON.

[11] Pandurengan RK, Dumont AG, Araujo DM (2010). Survival of patients with multiple primary malignancies: a study of 783 patients with gastrointestinal stromal tumor. Ann Oncol.

[12] Ikubo A, Matsufuji S, Morifuji Y (2019). Clinical features, prognosis, diagnostic approaches and treatment of multiple primary malignancies in the digestive system. Anticancer Res.

[13] Kohlmann W, Gruber S (2018). Microsatellite instability (MSI) testing. GeneReviews [Internet].

[14] Ichikawa D, Takahashi T, Hashimoto N (1996). Multiple primary cancers with microsatellite instability: report of a case. Jpn J Cancer Res.

[15] Yamashita K, Arimura Y, Kurokawa S (2000). Microsatellite instability in patients with multiple primary cancers of the gastrointestinal tract. Gut.

[16] Hong YS, Kim SY, Lee JS (2019). Oxaliplatin-based adjuvant chemotherapy for rectal cancer after preoperative chemoradiotherapy (ADORE): long-term results of a randomized controlled trial. J Clin Oncol.

[17] Smyth EC, Wotherspoon A, Peckitt C (2017). Mismatch repair deficiency, microsatellite instability, and survival: an exploratory analysis of the Medical Research Council Adjuvant Gastric Infusional Chemotherapy (MAGIC) trial. JAMA Oncol.

[18] Choi YY, Kim H, Shin SJ (2019). Microsatellite instability and programmed cell death-ligand 1 expression in stage II/III gastric cancer: post hoc analysis of the CLASSIC randomized controlled study. Ann Surg.

[19] Pierko J, Łukaszewicz J, Sawicka-Pierko A, Hady HR, Dadan J (2012). Synchronous gastric and rectal cancer in a 50 year-old man - case report. Pol Przegl Chir.

[20] Kwek BE, Ang TL, Fock KM, Teo EK (2011). Synchronous multifocal early gastric cancers and rectal adenocarcinoma: lesson learnt from image-enhanced endoscopy. J Dig Dis.

